# Supplementary material for: Splice-Junction-Based Mapping of Alternative Isoforms in the Human Proteome
Source: Cell Rep. Author manuscript; Available in PMC 2020 Jan 15. (PMC6961840; doi:10.1016/j.celrep.2019.11.026)
Supplement: 3 [file NIHMS1546469-supplement-3.zip › DF2/PXD006675/LeftVentricle-6-O75112-SRPQASSYSPAVAASSAPATHTSYSEGPAAPAPK.pdf]

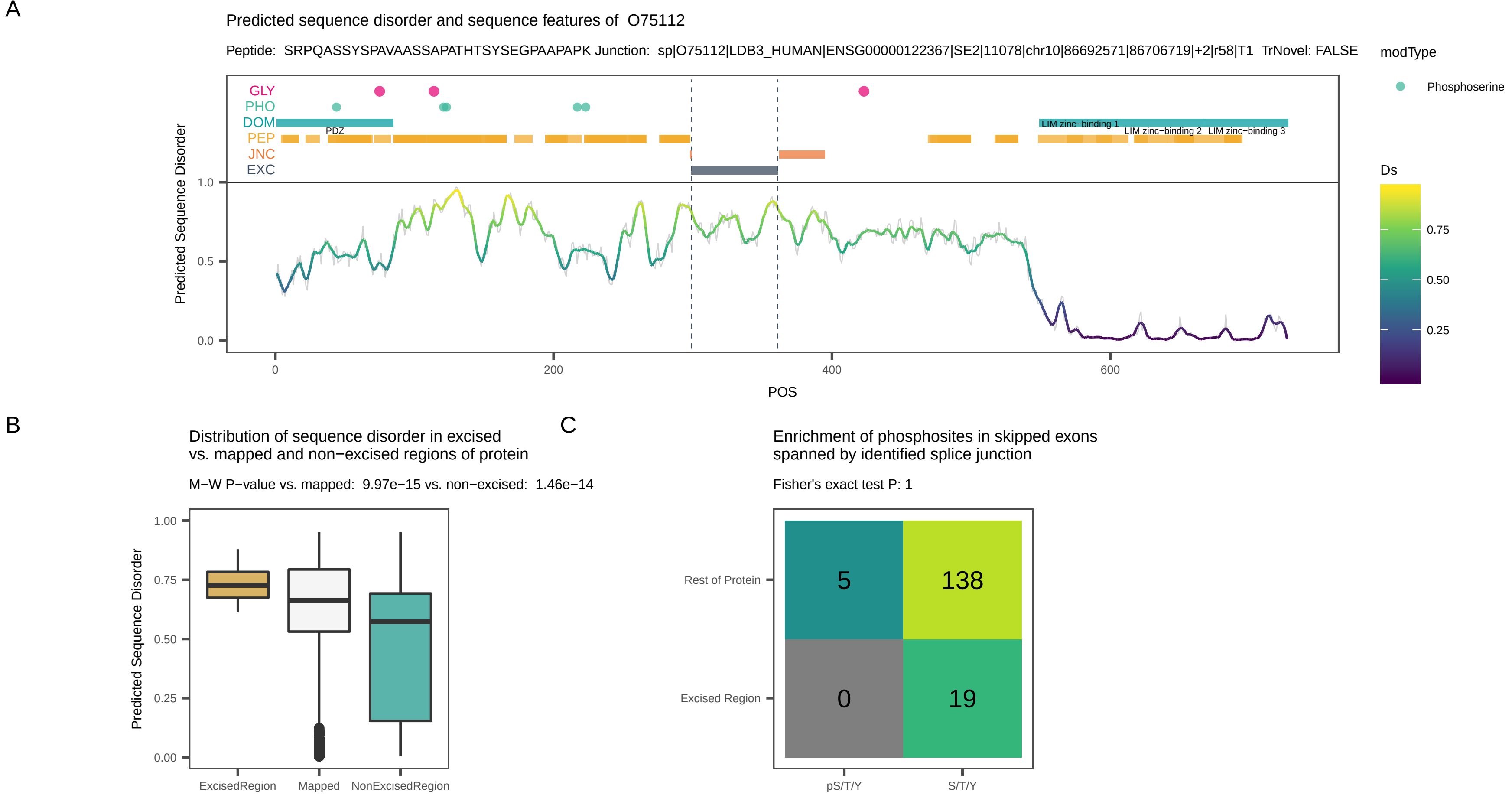
A
Predicted sequence disorder and sequence features of O75112
Peptide: SRPQASSYSPAVAASSAPATHTSYSEGPAAPAPK Junction: sp|O75112|LDB3_HUMAN|ENSG00000122367|SE2|11078|chr10|86692571|86706719|+2|r58|T1 TrNovel: FALSE
GLY
PHO
DOM
PEP
JNC
EXC
PDZ
LIM zinc−binding 1
LIM zinc−binding 2
LIM zinc−binding 3
Predicted Sequence Disorder
1.0
0.5
0.0
0
200
400
600
POS
modType
Phosphoserine
Ds
0.75
0.50
0.25
B
Distribution of sequence disorder in excised
vs. mapped and non−excised regions of protein
M−W P−value vs. mapped: 9.97e−15 vs. non−excised: 1.46e−14
Predicted Sequence Disorder
1.00
0.75
0.50
0.25
0.00
ExcisedRegion
Mapped
NonExcisedRegion
C
Enrichment of phosphosites in skipped exons
spanned by identified splice junction
Fisher's exact test P: 1
Rest of Protein
Excised Region
5
138
0
19
pS/T/Y
S/T/Y
